# Oligometastatic Esophagogastric Cancer: Does It Exist and How Do We Treat It?

**DOI:** 10.1007/s11912-024-01625-3

**Published:** 2025-01-03

**Authors:** Tiuri E. Kroese, Sebastiaan F. C. Bronzwaer, Peter S. N. van Rossum, Hanneke W. M. van Laarhoven, Richard van Hillegersberg

**Affiliations:** 1 Department of Radiation Oncology, University Hospital Zürich, University of Zurich, Rämistrasse 100, Zürich, 8091 Switzerland; 2 Department of Surgery, University Medical Center Utrecht, Utrecht University, Utrecht, The Netherlands; 3 Department of Radiation Oncology, Amsterdam UMC, Location VUmc, Amsterdam, The Netherlands; 4 Department of Medical Oncology, Amsterdam UMC Location University of Amsterdam, Amsterdam, The Netherlands; 5 Cancer Center Amsterdam, Cancer Treatment and Quality of Life, Amsterdam, The Netherlands

**Keywords:** Oligometastasis, Oligometastatic disease, Esophageal cancer, Gastric cancer, Metastasectomy, Stereotactic radiotherapy

## Abstract

**Purpose of the Review:**

This narrative review aims to provide an overview of recently completed randomized trials and expert consensus recommendations, and their implications for clinical practice and future trial design in patients with de-novo esophagogastric oligometastatic disease (OMD).

**Recent Findings:**

The IKF-575/RENAISSANCE phase III trial showed no significant overall survival difference between systemic therapy alone and systemic therapy combined with local therapy for patients with gastric or gastroesophageal junction cancer and de-novo OMD, except for patients with retroperitoneal lymph node metastases only. The ESO-Shanghai 13 phase II trial demonstrated superiority of adding local therapy to systemic therapy for progression-free and overall survival in oligometastatic esophageal squamous cell carcinoma. The OMEC project developed a multidisciplinary European consensus for OMD, proposing a restrictive definition of OMD. Clinical trial assessing the optimal treatment of care are urgently needed.

**Summary:**

The findings highlight the importance of strict patient selection for local metastasis-directed treatment and the need for stratifying patients based on histology and location of metastases. Future research should focus on identifying biomarkers and clinical features to guide multidisciplinary treatment approaches for OMD

## Introduction

Oligometastatic disease (OMD) refers to a type of metastatic disease characterized by limited metastatic spread (“oligo” = few, little) [1, 2]. The concept of OMD was first introduced by Hellman and Weichselbaum in 1995 [1]. OMD refers to a state between localized disease and polymetastatic disease, in which it is suggested that patients might benefit from local treatment of the limited metastases in addition to systemic therapy to improve survival [1, 2].

**Fig. 1:**
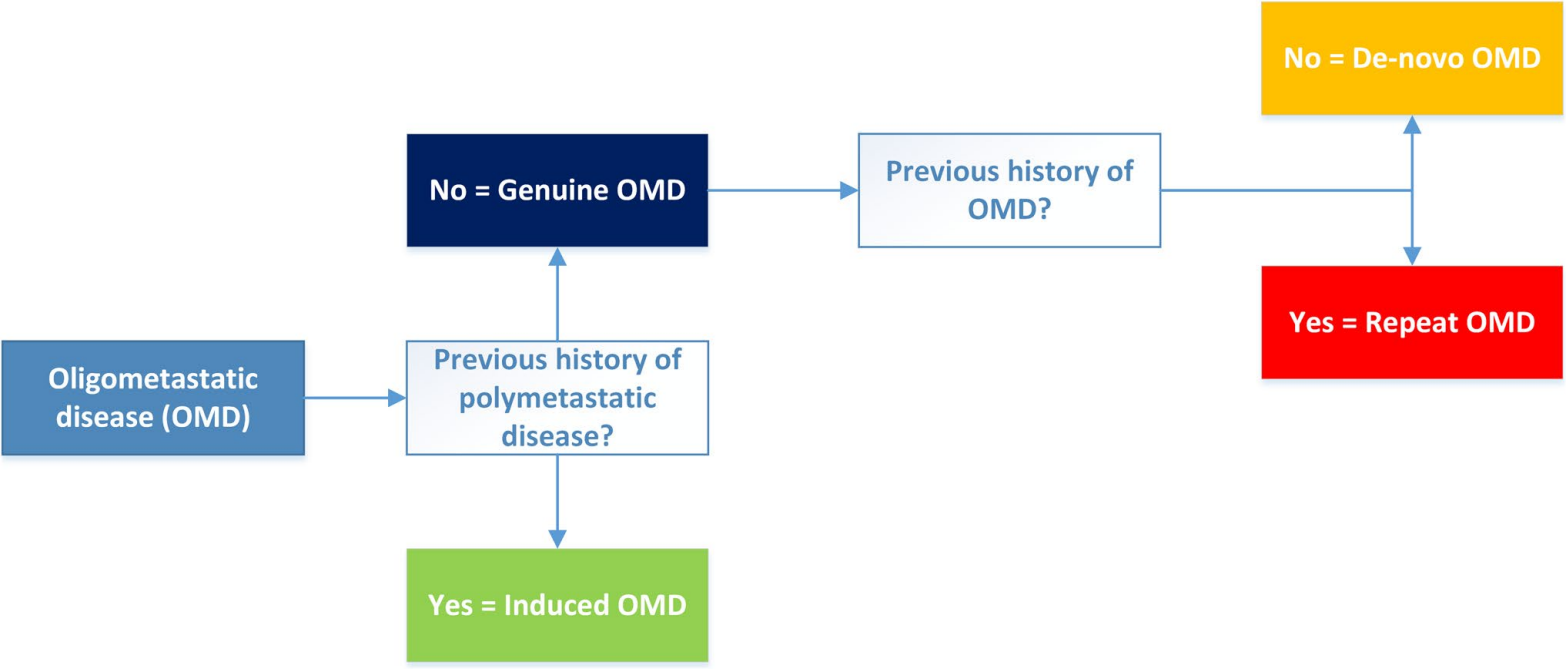
Types of OMD, adapted from Guckenberger et al. [3]

 The European Society for Radiotherapy and Oncology (ESTRO) and the European Organization for Research and Treatment of Cancer (EORTC) developed a classification for OMD in 2020 [3]. De-novo OMD is defined as the first time diagnosis of OMD without a previous history of OMD or polymetastatic disease. Other types of OMD are repeat OMD, i.e. OMD with a previous history of OMD, and induced OMD i.e. OMD with a previous history of polymetastatic disease [3]. Figure 1 provides an overview of the various types of OMD (adapted from Guckenberger et al.) [3].

To date, four non-randomized trials have showed survival benefits after local therapy for OMD in patients with esophagogastric cancer [4–7]. Two studies from China analyzed patients with esophageal squamous cell carcinoma and investigated the value of stereotactic body radiation therapy (SBRT) for de-novo OMD [4, 5]. Median overall survival for patients with esophageal squamous cell carcinoma who underwent SBRT was 12.8 months in the study by Zhao et al. [5] and 24.6 months in the study by Liu et al. [4] respectively. Additionally, one German study [6] and one Chinese study [7] included patients with oligometastatic adenocarcinoma of the stomach, investigated the value of metastasectomy for OMD. In the German study by Al-Batran et al., the median overall survival was 31.3 months for patients who responded to induction chemotherapy and who proceeded to surgery, versus 15.9 months for patients who did not respond to systemic therapy and who did not proceeded to surgery [6]. In the Chinese study by Cui et al., the median follow-up time was 30.0 months, and the median overall survival was not reached for the local therapy group plus systemic therapy versus 18.0 months for the systemic therapy alone group [7]. Finally, some studies are still underway [8–15].

 Recently, results of randomized controlled trials became available [16, 17]. The ESO-Shanghai 13 trial investigated the addition of local treatment to systemic therapy in patients with esophageal squamous cell carcinoma and metachronous de-novo OMD, and the IKF-575/RENAISSANCE (preliminary results) trial investigated the addition of surgical resection after chemotherapy for patients with adenocarcinoma the stomach and esophagogastric junction and synchronous de-novo OMD [16, 17]. This narrative review aims to provide an overview of recently completed randomized trials and expert consensus recommendations, and their implications for clinical practice and future trial design in patients with de-novo esophagogastric OMD.

## Completed Randomized Controlled Trials

### ESO-Shanghai 13

In the Chinese phase II ESO-Shanghai 13 trial, 116 patients over 18 years old with an ECOG performance status of 0–1 and confirmed metachronous oligometastatic squamous cell carcinoma of the esophagus were enrolled between March 2019 and September 2021 [16]. Patients had a controlled primary tumor through surgery or radiotherapy with no progression for at least 3 months [16]. De-novo OMD was characterized as having ≤ 4 metastatic sites in ≤ 3 different organs, each lesion measuring ≤ 5 cm, and all being suitable for local treatment without previous intervention [16]. Patients who had received antitumor treatments within the last 3 months or had polymetastatic disease were excluded from the trial [16].

The study randomized 104 patients 1:1 to receive either standard-of-care systemic therapy alone or combined systemic and local therapy. Randomization was balanced on 3 prognostic factors: number of disease sites (1 vs. 2–4), prior systemic therapies (first-line vs. second-line), and metastases location (non-regional lymph nodes vs. visceral metastases) [16]. Both groups underwent 4 cycles of standard chemotherapy, with an option for anti-PD-1 immunotherapy starting July 2020 [16]. The local therapy group, which included 20 of 53 patients receiving immunotherapy, had radiotherapy as the preferred treatment of choice, using stereotactic ablative body radiotherapy (SABR) in 38%, intensity-modulated (conventional) radiotherapy in 47%, and hypofractionated radiotherapy in 4% [16] SABR often entailed ≤ 5 fractions of ≥ 7 Gy, while conventional radiotherapy used ≥ 45–66 Gy at 1.8–2.0 Gy per fraction [16]. Other local treatments included surgery or thermal ablation [16]. In the systemic therapy group, local treatments were restricted to cases of disease progression, symptom relief, or palliative care [16].

The primary study endpoint was progression-free surviva, with secondary endpoints including overall survival, local control, toxicity, and quality of life [16]. The study showed a significant survival benefit for the group receiving combined therapy, with a median progression-free survival of 15.3 months compared to 6.4 months in the systemic-only group (stratified hazard ratio [HR] 0.26, 95% CI 0 16–0.42; *p* < 0.0001) [16]. This improvement in progression-free survival also led to an improved overall survival, where the median overall survival was not reached in the combined therapy group versus 18.6 months in the systemic therapy-only group (HR 0.42, 95% CI 0.24–0.74; *p* = 0.002) [16]. Notably, the addition of local therapy seemed less beneficial for patients receiving immunotherapy (HR for progression-free survival 0.49 [0.24–0.99]; log-rank *p* = 0·044 and HR for overall survival 0.57 [95% CI 0.24–1·36]; *p* = 0.19) [16]. The pattern of treatment failure also varied, with a higher occurrence of new lesions in the combined therapy group but significantly better local control (HR 0.11, 95% CI 0.05–0.24; *p* < 0.0001) [16]. The time until new lesions appeared was longer in the combined therapy group (16.8 months) compared to the systemic-only group (12.3 months; *p* = 0.0075) [16].

### IKF-575/RENAISSANCE Phase III Trial

In the German IKF-575/RENAISSANCE phase III trial, presented at ASCO 2024, 182 patients aged at least 18 years, with an ECOG performance status of 0–1 and histologically confirmed synchronous de-novo OMD from adenocarcinoma of the stomach or gastroesophageal junction were included between February 2016 and May 2024 [17]. De-novo OMD was defined as retroperitoneal lymph node metastases only or/and peritoneal carcinomatosis, ≤ 5 liver metastases, unilateral lung involvement, uni- or bilateral ovarian metastases, uni- or bilateral adrenal gland metastases, extra-abdominal lymph node metastases, or localized bone involvement [17].

A total of 139 patients were randomized 1:1 to standard-of-care systemic therapy combined with local therapy for all metastases (Arm A) or systemic therapy alone (Arm B) [17]. Patients were balanced by three prognostic factors: primary tumor location (gastric cancer vs. gastroesophageal junction), response to systemic therapy (complete response/partial response vs. stable disease), and location of the metastases (retroperitoneal lymph node metastases only vs. visceral metastases) [17].

Systemic therapy for both groups consisted of 4 cycles of standard-of-care fluorouracil, leucovorin, oxaliplatin and docetaxel (FLOT) chemotherapy [17]. Post-randomization, patients without disease progression were randomized into two arms [17]. Arm A received surgery followed by 4–8 cycles of FLOT chemotherapy, while Arm B received 4–8 cycles of FLOT chemotherapy alone [17].

The primary endpoint of the study was overall survival, with secondary endpoints including the proportion of patients with local control, toxicity, and quality of life. Pre-specified subgroup analyses were performed for patients with (1) retroperitoneal lymph node metastases only, (2) liver metastases, and (3) peritoneal metastases [17].

Patients in both groups were well balanced in baseline characteristics, including performance status and TNM stage [17]. Both groups received a similar number of pre-randomization chemotherapy cycles [17]. Surgery was performed in 91% of patients in Arm A, but also in 21% of patients in Arm B. In Arm A, complete resection of both the primary tumor and metastases was achieved in 56% of patients with retroperitoneal node metastases and 53% of patients with visceral metastases [17]. However, there was a difference in the number of received cycles of post-randomization chemotherapy) [17].

The study demonstrated that the cohort receiving systemic and local therapy (Arm A) had comparable overall survival to the systemic therapy-only cohort (Arm B) [17]. Median overall survival was 18.5 months (interquartile range [IQR]: 9.5–65.2) in Arm A versus 23.6 months (IQR: 14.0–41.2) in Arm B (*p* = 0.861) [17]. A benefit of additional local therapy was observed in patients with retroperitoneal lymph node metastases only, with a median overall survival of 29.6 months versus 17.1 months (Arm A compared with Arm B) [17]. Patients with liver metastases had comparable overall survival (24.9 versus 25.7 months), while those with peritoneal metastases had worse overall survival in Arm A compared to Arm B (11.9 vs. 18.6 months) [17]. After the end of study treatment, there was a difference in proportion of patients receiving further anticancer therapy (52% in arm A versus 82% in arm B) [17].

In summary, ESO-Shanghai 13 and RENAISSANCE report conflicting results on the benefit of local treatment for de-novo OMD. These conflicting results can be explained by several key differences between the studies [16]. Firstly, the primary tumor type and histology: the ESO-Shanghai study [16] included only patients with esophageal squamous cell carcinoma, whereas the IKF-575/RENAISSANCE trial [17] included patients with gastric or gastroesophageal junction adenocarcinoma. Secondly, the type of OMD: the ESO-Shanghai study included patients with metachronous de-novo OMD, had a lower cut-off for the number of metastases (4) and did not include patients with peritoneal metastases, whereas the IKF-575/RENAISSANCE trial included patients with synchronous de-novo OMD, had a higher cut-off for the number of metastases (5) and included patients with peritoneal metastases [16, 17]. Thirdly, the treatment of OMD: the ESO-Shanghai study predominantly used radiotherapy (SABR), [16] while the IKF-575/RENAISSANCE trial [17] used surgery for the primary tumor and metastases. In addition, it is important to note that a complete resection of the primary tumor and metastases was achieved in 56% of patients with retroperitoneal node metastases only and 53% of patients with visceral metastases in the IKF-575/RENAISSANCE trial [17].

## Recommendations from the OMEC Project

The OligoMetastatic Esophagogastric Cancer (OMEC) project was a multidisciplinary consensus project involving 49 expert centers across 16 European countries, aimed at developing a multidisciplinary consensus statement for the definition, diagnosis, and treatment of de-novo esophagogastric OMD [18]. The project included a systematic review of definitions of OMD and outcomes after local treatment for OMD [19], real-life clinical case discussions by expert centers in Europe [20], and a Delphi consensus study consisting of 2 online questionnaire rounds and a consensus meeting [21]. The resulting clinical practice guidelines [22] will be followed by a planned clinical prospective study.

In the OMEC project, a more restrictive definition of OMD was developed compared to the IKF-575/RENAISSANCE [17] or ESO-Shanghai trial [16]. In OMEC, de-novo OMD was defined by expert consensus as a maximum involvement of 1 organ with ≤ 3 metastases or 1 extra-regional lymph node station [22]. Notably, peritoneal metastases were excluded [18, 22].

Findings from the IKF-575/RENAISSANCE trial [17] support this more restrictive OMEC approach to defining OMD, showing that only the subgroup of patients with very limited metastases (i.e. retroperitoneal lymph node metastases only) benefited from radical local treatment of OMD. The group with liver metastases did not benefit from local treatment, but this group also included patients with ≤ 5 liver metastases or retroperitoneal lymph node metastases in addition to liver metastases [17]. Table 1 highlights the differences between the various definitions of de-novo OMD.

**Table 1:** Differences between definition, diagnosis and treatment of OMD according to IKF-575/RENAISSANCE [16], ESO-Shanghai 13 [17], and OMEC [22]

Study	Primary tumor	Number of metastases	Number of locations with metastases	Peritoneal metastases included	Type of OMD	Treatment
ESO-Shanghai 13 [16]	Esophageal SCC	≤ 4	≤ 3	No	Metachronous	ChT + RT vs. ChT
IKF-575/RENAISSANCE [17]	Gastric AC	≤ 5	≤ 2 (visceral *and* retroperitoneal)	Yes	Synchronous	ChT + Surgery vs. ChT
OMEC [22]	Esophageal AC and SCC and Gastric AC	≤ 3	1 (visceral *or* extra-regional lymph nodes)	No	Synchronous / Metachronous	ChT + local treatment for OMD

*AC *adenocarcinoma, *ChT *chemotherapy, *RT *radiotherapy, *SCC *squamous cell carcinoma.

**Fig. 2:**
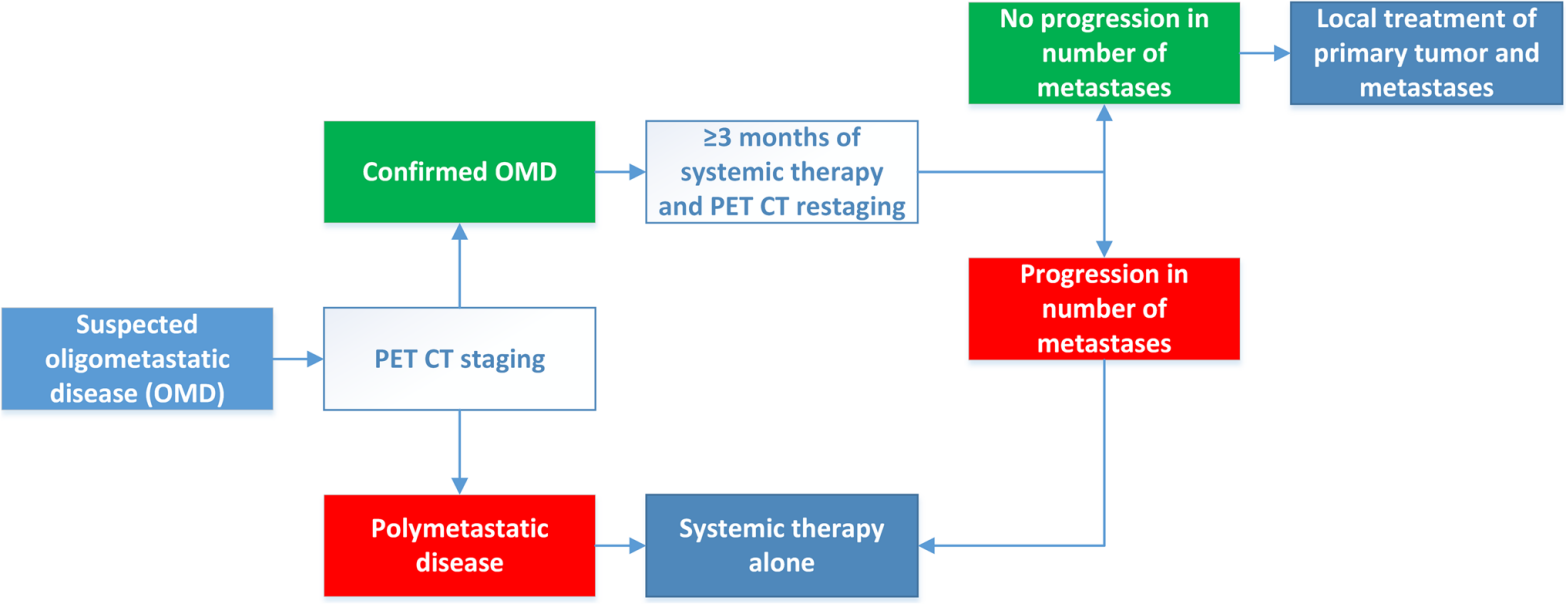
An overview of the treatment protocol according to OMEC [22]

According to OMEC, patients with suspected OMD would initially undergo ^18^F fluorodeoxyglucose positron emission tomography (^18^F-FDG PET/CT) to rule out polymetastatic disease. Those with a confirmed diagnosis of de-novo OMD would start with systemic therapy for ≥ 3 months. If, upon ^18^F-FDG PET/CT restaging, there is no progression in the number of metastases, local treatment of the metastases and the primary tumor (for synchronous OMD) would be administered. Figure 2 provides an overview of a recommended treatment strategy according to OMEC.

Local treatment for the primary tumor should be performed according to current international guidelines. Local treatment for metastases consist of either surgery, radiotherapy (SBRT) or radiofrequency ablation and should be performed according to the preference of the local multidisciplinary team. The local multidisciplinary team should also have the possibility to refer the patient to an expert center.

## Future Research

The findings of the ESO-Shanghai 13 [16] and IKF-575/RENAISSANCE [17] highlight the importance of patient selection and the need for stratifying patients based on the type and location of metastases. Future research should focus on identifying biomarkers or clinical features that can predict which patients will benefit most from local therapies such as those with retroperitoneal lymph node metastases as observed in the IKF-575/RENAISSANCE [17] trial. In addition, the results of the ESO-Shanghai 13 study [16] suggest that combining systemic therapy with local treatment, including advanced radiotherapy techniques, can improve outcomes. Future research should explore optimal combinations and sequences of systemic and local therapies. Finally, the OMEC project’s restrictive definition of OMD provides a framework for future clinical trials and practice. Prospective studies should validate these guidelines.

## Conclusions

OMD refers to a type of metastatic disease with limited metastatic spread. Recently, a consensus classification by ESTRO and EORTC defined three types of OMD: induced OMD, de-novo OMD, and repeat OMD. In the past year, the first 2 randomized controlled trials in oligometastatic esophagogastric cancer have been completed. The ESO-Shanghai 13 study highlighted the superiority of combined systemic and local therapy in progression-free and overall survival for patients with esophageal squamous cell carcinoma and metachronous de-novo OMD. The IKF-575/RENAISSANCE phase III trial showed no significant overall survival benefit of adding radical surgery for patients with gastric or gastroesophageal junction cancer and synchronous de-novo OMD, except for those with retroperitoneal lymph node metastases only. The OMEC project developed a multidisciplinary European consensus for esophagogastric OMD, proposing a rather restrictive definition of OMD and a treatment algorithm. This definition and treatment algorithm warrants validation in a prospective study.

## Key References

- Guckenberger M, Lievens Y, Bouma AB, et al. Characterisation and classification of oligometastatic disease: a European Society for Radiotherapy and Oncology and European Organisation for Research and Treatment of Cancer consensus recommendation. *The Lancet Oncology*. 2020;21(1):e18-e28. 10.1016/S1470-2045(19)30718-1.
  - ⚬ Important consensus document for the definition of OMD.
- Al-Batran SE, Homann N, Pauligk C, et al. Effect of neoadjuvant chemotherapy followed by surgical resection on survival in patients with limited metastatic gastric or gastroesophageal junction cancer: The AIO-FLOT3 trial. *JAMA Oncology*. 2017;3(9):1237–1244. 10.1001/jamaoncol.20170515.
  - ⚬ Prospective non-randomized trial in the field of esophagogastric OMD.
- Cui Y, Yu Y, Zheng S, et al. Does resection after neoadjuvant chemotherapy of docetaxel, oxaliplatin, and S-1 (DOS regimen) benefit for gastric cancer patients with single non-curable factor? a multicenter, prospective cohort study (Neo-REGATTA). *BMC Cancer*. 2023;23(1). 10.1186/s12885-023-10773-x.
  - ⚬ Prospective non-randomized trial in the field of esophagogastric OMD.
- Liu Q, Zhu Z, Chen Y, et al. Phase 2 Study of Stereotactic Body Radiation Therapy for Patients with Oligometastatic Esophageal Squamous Cell Carcinoma. *International Journal of Radiation Oncology Biology Physics*. 2020;108(3):707–715. 10.1016/j.ijrobp.2020.05.003.
  - ⚬ Prospective non-randomized trial in the field of esophagogastric OMD.
- Zhao W, Ke S, Cai X, et al. Radiotherapy plus camrelizumab and irinotecan for oligometastatic esophageal squamous cell carcinoma patients after first-line immunotherapy plus chemotherapy failure: An open-label, single-arm, phase II trial. *Radiotherapy and Oncology*. 2023;184. 10.1016/j.radonc.2023.109679.
  - ⚬ Prospective non-randomized trial in the field of esophagogastric OMD.
- Liu Q, Chen J, Lin Y, et al. Systemic therapy with or without local intervention for oligometastatic oesophageal squamous cell carcinoma (ESO-Shanghai 13): an open-label, randomised, phase 2 trial. *The Lancet Gastroenterology & Hepatology*. 2023. 10.1016/S2468-1253(23)00316-3.
  - ⚬ Randomized controlled trial in the field of esophagogastric OMD.
- Al-Batran SE. Effect of chemotherapy/targeted therapy alone vs. chemotherapy/targeted therapy followed by radical surgical resection on survival and quality of life in patients with limited-metastatic adenocarcinoma of the stomach or esophagogastric junction: The IKF-575/RENAISSANCE phase III trial. presented at: ASCO; 2024;
  - ⚬ Randomized controlled trial in the field of esophagogastric OMD.
- Kroese TE, Bronzwaer S, van Rossum PSN, et al. European clinical practice guidelines for the definition, diagnosis, and treatment of oligometastatic esophagogastric cancer (OMEC-4). *European Journal of Cancer*. 2024;204. 10.1016/j.ejca.2024.114062.
  - ⚬ Important consensus document for the definition of esophagogastric OMD.

## Data Availability

No datasets were generated or analysed during the current study.
